# Biopsychosocial factors in oral and systemic diseases: a scoping review

**DOI:** 10.3389/froh.2024.1378467

**Published:** 2024-05-30

**Authors:** Abby L. J. Hensel, Kathryn Nicholson, Kelly K. Anderson, Noha A. Gomaa

**Affiliations:** ^1^ Dentistry, Schulich School of Medicine & Dentistry, Western University, London, ON, Canada; ^2^ Epidemiology and Biostatistics, Schulich School of Medicine & Dentistry, Western University, London, ON, Canada; ^3^ Psychiatry, Schulich School of Medicine & Dentistry, Western University, London, ON, Canada; ^4^ Children's Health, Lawson Health Research Institute, London, ON, Canada

**Keywords:** psychological stress, oral disease, systemic disease, psychosocial factors, cortisol, aging, lifespan, biopsychosocial factors

## Abstract

**Background:**

The association between chronic oral diseases and other major systemic health conditions, commonly referred to as the oral-systemic health connection, has been previously studied with several underlying common risk factors and pathways linking both groups of diseases. Psychosocial factors contribute to an increased susceptibility to chronic oral and non-oral diseases. The aim of this review is to summarize the current state of knowledge on the role of psychosocial stress in chronic oral and systemic diseases.

**Methods:**

A search strategy was built and a literature search was conducted using four databases (CINAHL, Embase, Medline, PsycINFO). A combination of search terms related to psychosocial stress, systemic disease, and oral conditions were used. Studies were eligible for inclusion if they included human adults (aged 18 years and older), included psychosocial factors as an exposure measure, and outcome measures of both an oral and systemic condition. Only English-language articles were considered. Pilot testing of the data extraction form and calibration were conducted and data were extracted independently by one researcher.

**Results:**

A total of fifteen articles out of eighty full-text articles screened were determined to be eligible for inclusion in this review. Periodontal disease was the most commonly studied oral disease, measured in 53% of included articles, with the most commonly studied systemic diseases being of mental health conditions (40%) and diabetes (47%). Psychosocial stress was measured using a range of psychometric indicators and/or biomarkers, including perceived stress, individual behaviours, childhood adversity, and cortisol. In total, fourteen studies found a positive association between measures of psychosocial stress and oral-systemic health.

**Conclusion:**

Psychosocial stress may be a common contributor to both chronic oral and non-oral diseases.

## 1 Introduction

The oral-systemic disease or the link between oral and other health conditions continues to be a dynamic area of interest and relevance to dental practice and policy (1). This relationship can be bidirectional where various systemic diseases and related medications can have oral manifestations, while oral pathologies can have a systemic impact (2, 3). Studies, theories and postulations about how oral and systemic diseases are linked have been ongoing for several decades (2, 4). Through the focal infection theory, defined more than a century ago, W.D. Miller suggested the mouth as a source of infection, theorizing that oral microorganisms and/or their products are able to access other areas and organs that are adjacent to or distant from the oral cavity (5, 6). More recently, research on the oral-systemic disease connection has considerably intensified and evolved, and there is now a plethora of studies that demonstrate that both groups of diseases are linked through social and biological pathways. For example, periodontal disease has been consistently associated with atherosclerosis, heart disease, diabetes, osteoporosis, and other conditions through an underlying inflammatory process (7–12). The number of functional teeth has also been suggested as a predictor of mortality and cognitive health in older adults (13, 14). Importantly, both groups of diseases are known to stem from adverse social exposures such as precarious living conditions, poor social support and the inability to afford medical and/or dental care (15). Therefore, deciphering the pathways that link these common social exposures to disease outcomes is important to identify “mid-stream” and modifiable points of intervention (16).

The biomedical model, which focuses on health purely in terms of biological factors (17–19), has traditionally be used to explain the oral-systemic disease connection and has dominated discussions in this area. However, the biomedical model is often criticized due to the biomedical approach treating independent organs or systems in isolation may alleviate some symptoms without solving the “root cause” of the condition (17, 20, 21). In doing so, the biomedical model reduces an individual to an object of illness rather than as an active part of the healing process (22). Alternatively, to address the limitations and criticisms of the biomedical model, George Engel (1977) proposed a new model in the 1970s to better understand the various factors involved in health and disease (23). The biopsychosocial model is commonly used to explain how biological (e.g., viruses, bacteria, immune responses, etc.), psychological (e.g., stress, coping strategies, health behaviours, etc.), and social (e.g., race/ethnicity, level of education, employment, etc.) factors work to influence and shape health across the lifespan (24–27). It also acknowledges modern medical advances while also emphasizing that many illnesses cannot be understood by looking for changes at the cellular or molecular levels (24, 25, 28, 29).

Following the biopsychosocial approach, social and psychosocial exposures are known as common risk factors and pathways to oral and systemic health conditions (30). According to the American Psychological Association (APA), a psychosocial stressor refers to “a life situation that creates an unusual or intense level of stress that may contribute to the development or aggravation of mental disorder, illness, or maladaptive behaviour” (31). Previous research has demonstrated that stress may alter the internal homeosis. The hypothalamic-pituitary-adrenal (HPA) axis is considered one of the major endocrine systems that maintains homeostasis (32, 33). The parvocellular neurons of the paraventricular nucleus of the hypothalamus secrete corticotropin releasing hormone (CRH) and arginine vasopressin (AVP) during periods of acute stress, which activates the HPA axis (34, 35). These neuropeptides activate the synthesis and release of adrenocorticotropin hormone (ACTH) from the pituitary (32–35). ACTH secretion then stimulates the adrenal cortex to synthesize glucocorticoids, such as cortisol, which helps regulate inflammatory responses and lymphocytic activity (32–35). Although short-term elevations in glucocorticoids can reduce inflammation and mobilize immune components, when these hormones are produced over an extended period of time under conditions of chronic stress, they may impair immune function by inhibiting the activity of immunoglobulin A (IgA), immunoglobulin G (IgG), and neutrophils (36). IgA antibodies reduce the initial colonization of periodontal organisms, and IgG antibodies may make periodontal pathogens more vulnerable to phagocytosis by neutrophils (36, 37). Therefore, depressed immunity and chronically elevated cortisol may contribute to in higher levels of inflammation and exacerbation of numerous physical diseases over the lifespan, including periodontitis, heart disease, and diabetes (36–43).

In addition to altering physiological responses, psychosocial stress can be linked to health-harming behaviours that lead to oral and systemic diseases (44, 45). For example, a recent study has shown that smoking, alcohol consumption, and less frequent tooth-brushing concentrated in more stressed individuals (46). Psychosocial stress has also been shown to impact stress-coping mechanisms and to dysregulate central autonomic functions involved (47, 48). The latter has demonstrated how social adversity links to altered structure and function of stress-regulating brain areas including the hippocampus and associated behavioural inclinations (49). Inadequate coping skills have also been shown to exacerbate the association between periodontal disease and diabetes (50), as well as stress and heart disease (51, 52).

The impact of social and psychosocial exposures on health outcomes may be influenced by the magnitude and the timing at which the exposure occurs over the lifespan (26, 53). The life-course model envisions current health to be shaped by earlier exposures to physical, environmental, and psychosocial factors that mold biological outcomes (54). According to the developmental theory of the life-course perspective (life-span development), which focuses on the role of critical and sensitive periods in shaping health overtime, adverse childhood experiences (ACEs) such as growing up in poverty and/or experiencing neglect or abuse may alter the regulation of the HPA axis and increase the risk for health problems that manifest later in life (54–57). Although responses to an adverse environment may initially appear adaptive, prolonged dysregulation of physiologic stress mechanisms can persist into adulthood and older age (54, 58). For example, adults who report ACEs have been shown to have worse oral health outcomes than those who did not report ACEs (59). ACEs such as abuse in its various forms (physical, psychological, emotional abuse and/or sexual) and neglect have been shown to be associated with oral health problems including tooth loss, dental pain, and periodontal diseases (59). Additionally, early life stress has been shown to increase the risk of diabetes (60), ischemic heart disease (61), stroke (61), and hypertension (62). Some studies have also demonstrated that there is a graded dose-response relationship between ACEs and health outcomes in which the risk of diseases increases as the number of ACEs which an individual has experienced rises (63).

To this end, we recognize that psychosocial stress may play a pivotal role in the disease process, with a magnitude of effect that can vary according to the timing of the exposure and age of the individual. However, knowledge of empirical studies that have examined the role of psychosocial stress at different timepoints in life and how it can contribute to both oral and systemic health connection needs delineation. Therefore, in this review, we aim to map and identify gaps in the literature relating to the role of psychosocial factors in the co-occurrence of chronic oral and systemic health conditions. First, we provide an overview of the pathways and risk factors that are common to both oral and systemic diseases. We then present our systematic search in which we summarize the role of psychosocial stress, assessed both biologically and psychometrically, in the oral-systemic disease connection to identify knowledge gaps and future research opportunities.

## 2 Methods

This scoping review aims to describe knowledge on the contribution of psychosocial factors to the co-existence of oral and systemic diseases in adults. A scoping review methodology was selected to examine the available literature, summarize findings, and identify knowledge gaps, and was conducted following the approach suggested by Arksey and O'Malley (64). Searches, data extraction, and reporting of results were conducted following the PRISMA-ScR (Preferred Reporting Items for Systematic Review and Meta-Analysis extension for Scoping Reviews) checklist (Supplementary File) (65). The central research question for this scoping review was: “What is the state of current knowledge on the role of biopsychosocial factors in the co-occurrence of oral and systemic diseases in adults?”. This was curated using the following parameters:

Population: Adults aged 18 years and older
Exposure: Psychosocial factors (e.g., psychological stress, life stress, cortisol)
Comparison: Adults without psychosocial stress
Outcomes: Oral health conditions (e.g., periodontal disease, dental caries) and systemic disease (e.g., asthma, dementia, stroke)

### 2.1 Search strategy

A systematic search of the literature was done using the following electronic databases: CINAHL, Embase, Medline, and PsycINFO in March 2022, and updated in March 2023. Individual search strategies were developed for each database to account for unique indexing terms. The search strategy for each database is shown in the Supplementary File. A combination of keywords and search terms using Boolean operators, truncation, phrase searching, and MeSH terms were used in the search strategy. These included: (i) psychosocial stress (e.g., psychological stress, adverse childhood experiences, life stress, (ii) oral health conditions (e.g., periodontal disease, dental caries, oral health), and (iii) systemic diseases were selected based on the chronic conditions that were consistent with the recommendation of the Public Health Agency of Canada (PHAC) (66). Systemic conditions search terms included arthritis, asthma, cancer, COPD, dementia, diabetes, heart disease, stroke. The search included relevant published and unpublished literature found in theses retrieved from online accessible university repositories available in the English language without other restriction on study design or article type. The reference lists and bibliographies of relevant studies were hand searched for further references.

### 2.2 Eligibility criteria, article selection, and data extraction

Titles and abstracts were reviewed using the following inclusion criteria: human empirical studies of adults (aged 18 years and above), psychosocial stress as an exposure measure, and outcome measures that included both an oral and systemic condition. Other criteria for inclusion were: (i) population-based observational studies; (ii) peer-reviewed publication to assure a minimal threshold for quality of the studies; (iii) no geographical restrictions were placed so as to allow for a global perspective; (iv) studies published in the English language. Studies were excluded if they were conducted in animals, assessed only inflammatory biomarkers in relation to stress without a disease outcome, conducted in children (0–17 years), or were COVID-19 related. The reference lists and bibliographies of relevant systematic reviews, meta-analyses, case-reports, overviews, and commentaries were scanned to identify relevant studies; however, the reviews themselves were excluded.

Search results were extracted to Endnote and duplicates of identified records were removed and screened before title/abstract/full-text assessment. One author (A.H.) then independently conducted the eligibility screening process based on titles, abstracts, and full text. To ensure reviewer consistency, the first 50 articles were re-screened for inclusion after all other articles. Records that passed the initial screening had their full text downloaded and key information extracted using a Microsoft Excel 2019 sheet (Microsoft Corporation, United States) to assess for inclusion. Data extraction form and the extraction categories were determined based on discussions between co-authors. Pilot testing of the data extraction form for calibration was conducted. Key information included: author(s) and year of publication; study design; study focus/aim; country of origin; sample size and demographics; exposure variable(s); outcome variables; and key findings. Results and key extracted information are reported in a narrative and table format following PRISMA-ScR guidelines. Consistent with the scoping review approach and as we report the results in narrative form, no critical appraisal of studies was conducted (65).

## 3 Results

**Figure 1:**
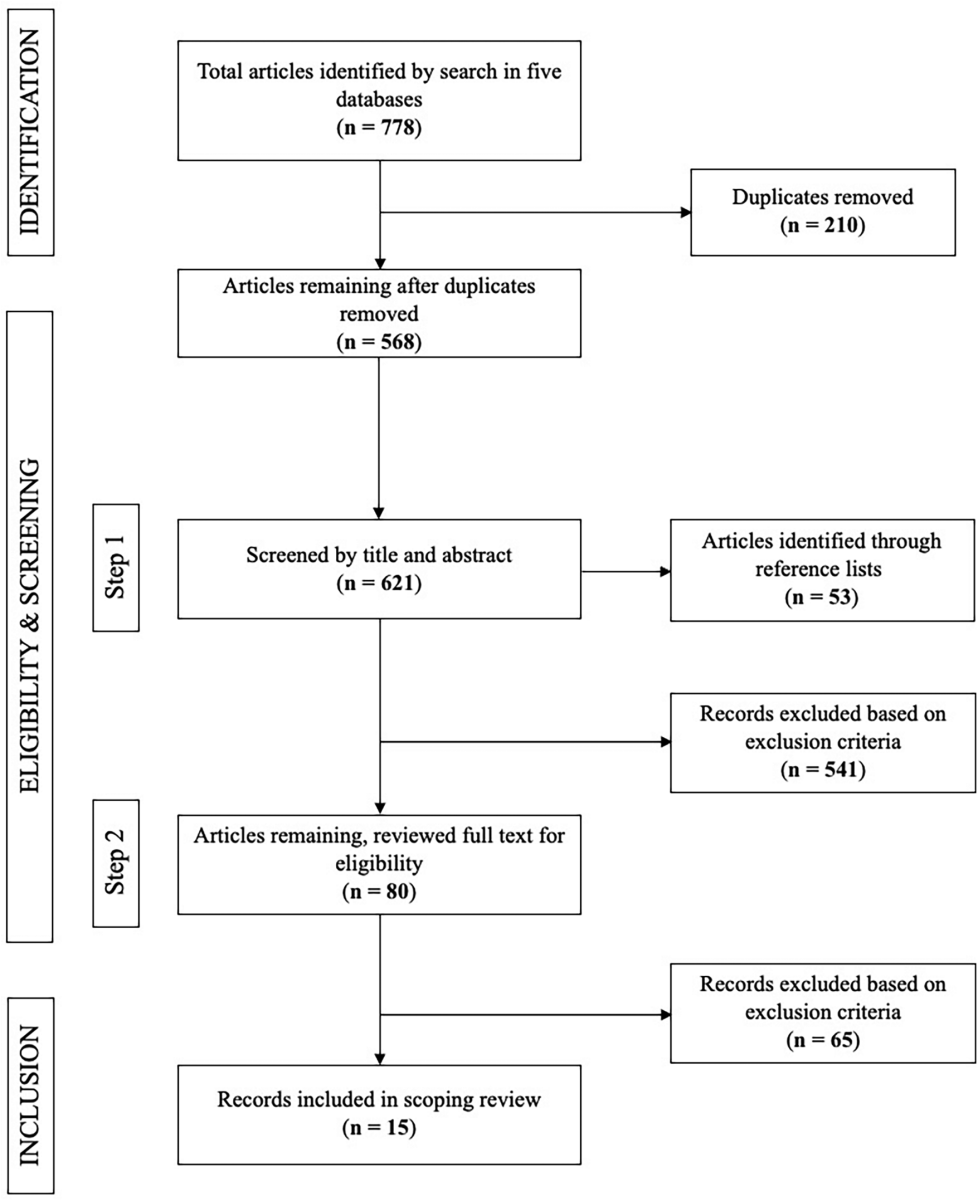
PRISMA flow diagram depicting the article selection process.

A total of 778 articles were identified and underwent titles/abstract screening for relevance. After screening by title and abstract, 556 articles were excluded. The most common reason for exclusion was assessing inflammatory biomarkers as an outcome, or not assessing oral and systemic conditions together. After evaluating the full text of 80 articles, a total of 15 studies met the inclusion criteria for the current review (Figure 1).

### 3.1 Characteristics of included studies

Of the fifteen articles included in this review, twelve studies used a psychometric measure of stress (Table 1) (67–78), five studies measured stress biologically via cortisol (Table 2) (72, 74, 79–81), and two studies assessed stress both psychometrically and biologically (72, 74). In total, the studies in this review involved 43,472 individuals, where the weighted mean age of participants was 60.0 years (range 17–82 years) and 45.9% were female. With regard to study design, six studies were of case-control design (69, 71–73, 79, 80), five were cross-sectional (67, 74–76, 81), three were cohort studies (68, 70, 78), and one was a clinical trial including baseline data comparing darapladib, a selective oral inhibitor of lipoprotein-associated phospholipase A_2_, with placebo in patients with coronary heart disease (77). There was also variation in geographical location, with four studies conducted in North America (67, 70, 74, 76), four in the Middle-East (68, 73, 79, 80), two in Europe (69, 71), three in Asia (72, 75, 81), one in New Zealand (78), and one multi-country study (77).

**Table 1:** Summary of studies using psychometric assessments for the role of psychosocial stress in co-existence of oral and systemic diseases.

Author, Year	Study design	Country	Sample size	% female	Mean age/age range (year)	Measure of stress	Oral condition	Systemic disease	Main finding
Perceived stress									
Albright et al. (67)	Cross-sectional	USA	88	52%	68.8	Experience of life stress (interview)	Poor oral health dental interview/exam	Diabetes (BMI)	Stress is associated with the presence of diabetes, obesity, and periodontal disease
Cinar & Schou (68)	Prospective intervention cohort	Turkey	186 (77 intervention, 109 controls)	NR	30–65	Perceived stress (self-reported), health coaching intervention	Tooth loss, CAL (clinical assessment)	Diabetes (HBA1C levels)	CAL and diabetes were associated with the higher stress control group
Kjellström et al. (69)	Case-control	Sweden	1,610 (805 cases with first MI, 805 controls)	19%	62 ± 8	Psychosocial stress (self-reported questionnaire)	Periodontitis (clinical examination and x-ray of bone loss)	Depression (MADRS), MI	Patients with a first MI experienced higher psychosocial stress and greater risk for depression and periodontitis
Lim et al. (70)	Prospective cohort	USA	1,021	96%	29	Emotional distress (self-reported)	Dental caries (clinical exam)	Self-reported heart condition, endocarditis, diabetes, epilepsy, heart attack	Individuals with emotional distress and chronic health conditions had increased risk of dental caries
Monteiro da Silva et al. (71)	Case-control	United Kingdom	150 (50 rapidly progressive periodontitis, 50 chronic periodontitis, 50 control)	66%	40.6	Perceived stress (PSS)	Periodontitis (clinical exam)	Depression (HADS)	Greater perceived stress and depression in patients with rapidly progressive periodontitis
Rajhans et al. (72)	Case-control	India	60 (20 chronic periodontitis with diabetes, 20 chronic periodontitis without diabetes, 20 controls)	NR	35–50	Perceived life stress (PSS)	Chronic periodontitis (clinical exam of PI GI, PD, CAL)	Diabetes (blood samples)	Perceived stress was higher in patients with both periodontitis and diabetes compared to healthy controls
Rezazadeh et al. (73)	Case-control	Iran	38 (19 cases, 19 controls)	16%	52	Perceived stress (DASS-21)	BMS (VAS)	Depression, (DASS-21), sleep disorder (PSQI)	Higher prevalence of stress, depression, and sleep disorders in BMS patients
Rosania et al. (74)	Cross-sectional	USA & Canada	45	69%	45–82	Chronic stress (DSP)	Periodontal disease, tooth loss, CAL (clinical exam)	Depression (CES-D)	Chronic stress, cortisol, and depression correlated with greater CAL, tooth loss, and periodontal disease
Living conditions and lifestyle behaviours									
Negishi et al. (75)	Cross-sectional	Japan	57	16%	17–79	Self-reported lifestyle (smoking, drinking, diet, exercise, financial problems, distress)	PD, alveolar bone loss (clinical exam)	Diabetes (HBA1C)	Drinking habits, anger, high levels of HbA1c associated with high alveolar bone loss and probing depth greater than 6 mm in diabetic patients
Parbhakar et al. (76)	Cross-sectional	Canada	23,131	51%	35+	Living conditions (sense of belonging, food security), individual behaviour (smoking, exercise)	Self-reported oral health	Self-reported arthritis, hypertension, COPD, diabetes, heart disease, stroke	Living conditions and individuals behaviours attenuate the association between oral and systemic diseases
Vedin et al. (77)	RCT	Global	15,828	19%	65	Lifestyle (education, stress, alcohol, exercise; self-reported)	Self-reported tooth loss, bleeding gums	Coronary heart disease (prior MI or prior coronary revascularization)	Lifestyle stress was associated with increased odds of tooth loss and bleeding gums in coronary heart disease patients
Childhood adversity									
Poulton et al., (78)	Cohort	New Zealand	980	48%	26	Socioeconomic disadvantage in childhood	Gingival bleeding, CAL, periodontal disease, dental caries (clinical assessment)	Cardiovascular health, depression (clinical diagnosis based on DSM-IV criteria)	Children with low socioeconomic status had increased adult incidence of poor cardiovascular health and oral health conditions

BMI, body mass index; CAL, clinical attachment loss; MADRS, Montgomery–Åsberg depression rating scale; MI, myocardial infarction; PSS, perceived stress scale; HADS, hospital anxiety and depression scale; PI, approximal plaque index; GI, gingival index; PD, probing depth; DASS, depression, anxiety, and stress scale; VAS, visual analog scale; PSQI, pittsburgh sleep quality index; DSP, derogatis stress profile; CES-D, center for epidemiologic studies depression scale; COPD, chronic obstructive pulmonary disease; RCT, randomized controlled trial; DSM-IV, diagnostic and statistical manual of mental disorders, 5th edition; NR, not reported.

**Table 2:** Summary of studies using biological assessments of psychosocial stress (i.e., cortisol) in the oral-systemic health connection.

Author, Year	Study design	Country	Sample size	% female	Mean age/age range (year)	Measure of stress	Oral condition	Systemic disease	Main finding
Albahli et al., (79)	Case-control	Saudi Arabia	40 (20 cases, 20 controls)	13%	39.1 ± 12.1; 38.3 ± 11.6	Salivary cortisol	Periodontal disease (clinical examination of GI, PI, PD, CAL)	Schizophrenia (clinical diagnosis)	Higher periodontal parameters and lower cortisol in schizophrenic patients
Rajhans et al., (72)	Case-control	India	60 (20 chronic periodontitis with diabetes, 20 chronic periodontitis without diabetes, 20 controls)	NR	35–50	Serum cortisol	Chronic periodontitis (clinical examination of PI GI, PD, CAL)	Diabetes (blood samples)	Clinical attachment levels and mean cortisol levels were highest in patients with both periodontitis and diabetes
Rosania et al., (74)	Cross-sectional	USA & Canada	45	69%	45–82	Salivary cortisol	Periodontal disease, tooth loss, CAL (clinical examination)	Depression (CES-D)	Chronic stress and cortisol correlated with greater CAL, tooth loss, and periodontal disease; depression associated with greater tooth loss
Salehi et al., (80)	Case-control	Iran	132 (44 diabetic, 44 prediabetic, 44 controls)	77.3%%	54 ± 5.7; 48 ± 8.7; 43 ± 11.9	Salivary cortisol	DMFT (clinical examination)	Diabetes (clinical diagnosis)	Salivary cortisol and DMFT was higher in diabetic patients; salivary cortisol was associated with DMFT index
Yang et al., (81)	Cross-sectional	China	106	38%	75.3 ± 6.6	Cortisol, inflammatory biomarkers	Dental caries, periodontal disease, self-reported oral health (clinical examination and self-reported)	Alzheimer's disease (clinical diagnosis)	Stress biomarkers and oral health indicators were poorer in individuals with Alzheimer's disease compared to those with subjective cognitive decline or mild cognitive impairment

GI, gingival index; PI, approximal plaque index; PD, probing depth; CAL, clinical attachment loss; CES-D, center for epidemiologic studies depression scale; DMFT, decayed, missing, and filled teeth; NR, not reported.

### 3.2 Oral and systemic diseases

Periodontal disease was the most common oral health outcome measured, included in 53% (*n* = 8) of all included studies (69, 71, 72, 74, 75, 78, 79, 81). The majority of studies (*n* = 12) recorded oral disease using clinical examinations or diagnosis of participants (80%) (67–75, 78–80), while the remaining three studies assessed oral health using self-report questionnaires (76, 77, 81). Clinically assessed outcomes included periodontal disease (69, 71, 72, 74, 75, 78, 79, 81), dental caries (70, 78, 81), tooth loss (68, 74, 77, 80), clinical attachment loss (68, 72, 74, 78, 79), burning mouth syndrome (73), and poor oral health (67, 76, 81). Self-reported outcomes included self-reported oral health (67, 76, 81), tooth loss (77), and bleeding gums (77). Diabetes (67, 68, 70, 72, 75, 76, 80), heart diseases (e.g., coronary heart disease, myocardial infarction) (70, 76–78), and mental health conditions (e.g., depression and schizophrenia) (69, 71, 73, 74, 78, 79) were the most common systemic diseases studied, comprising 47%, 27%, 40% of eligible studies, respectively. Other systemic diseases included hypertension (76), arthritis (76), sleep disorders (73), chronic obstructive pulmonary disorders (COPD) (76), and Alzheimer's disease (81). Four articles studied more than one systemic disease outcome (69, 70, 73, 76). Physical measures, such as BMI, blood samples, and clinical exams, were used to assess 60% of systemic disease outcomes (67, 68, 72, 75, 77–81), while self-report questionnaires, standardized assessment scales, and interviews were used in 40% of studies (69–71, 73, 74, 76).

Periodontal disease including gingival bleeding and clinical attachment loss were most commonly studied in relation to depression (69, 71, 74, 78) and diabetes (68, 72, 75). Self-reported oral health, dental caries and tooth loss were also studied in relation to heart disease (70, 76–78), diabetes (67, 68, 70, 76, 80) and depression (74, 78). Burning mouth syndrome was assessed in relation to depression (73). One study used only self-report questionnaires to assess both oral and systemic health outcomes (76), seven used only clinical examinations (67, 68, 72, 75, 79–81), and seven used a combination of self-report questionnaires and clinical exams (69–71, 73, 74, 77, 78) to assess oral and systemic health outcomes.

### 3.3 Psychosocial stress in oral and systemic diseases

Twelve articles (80%) included in this review measured stress psychometrically, investigating its association with oral and systemic diseases (67–78). Psychometric stress variables fell into four main categories: “perceived stress”, “lifestyle or individual behaviour”, and “childhood adversity”. Of the articles that assessed perceived stress (67–74), two studies employed Cohen's Perceived Stress Scale (PSS) (71, 72), one used the Depression, Anxiety and Stress Scale (DASS) (73), and one study used the Derogatis Stress Profile (DSP) (74). The remaining four perceived stress studies (67–70) interviewed participants, either in person or using questionnaires, with questions pertaining to experiencing life stress, such as “Have you ever been told by a doctor or other health professional that you had an anxiety or stress disorder?”. Lifestyle or individual behaviours were measured using questionnaires that focused on living conditions and behaviours and included questions on smoking, alcohol consumption, diet, distress, financial stress, etc. (75–77). Finally, childhood adversity was assessed in a cohort study using socioeconomic disadvantage in childhood (78).

Eight articles (53%) concluded that perceived life stress was higher in individuals with both oral and systemic disease outcomes (67–74). Higher prevalence of depression and perceived stress were found in both burning mouth syndrome (73) and periodontal disease (71, 74). Perceived stress was also found to be higher in individuals with both periodontal disease and diabetes when compared to healthy controls (68, 72). Participants with heart disease experienced greater psychosocial stress and demonstrated increased risk for periodontitis (69). Individuals with emotional distress and heart disease had a higher risk of developing dental caries (70). However, one study found no evidence for an association between psychological stress, depression, and periodontitis (73).

Poor living conditions, such as low income and food insecurity, increased the odds of the co-occurrence of oral-systemic disease (76), and negative lifestyle was associated with poor gingival health in diabetic patients (75) and increased the odds of tooth loss in coronary heart disease patients (77).

Only one study explored the association between childhood adversity and adult health (78). As part of a longitudinal cohort, health and behaviour of participants was assessed from birth until 26 years old (78). In this study, researchers found that children who grew up in families of lower socioeconomic backgrounds were at a greater risk of having poor cardiovascular health, depression, and periodontal disease in adulthood (78). However, we did not identify studies using an adverse childhood experiences (ACEs) score in association with oral and systemic diseases.

### 3.4 Biomarkers of psychosocial stress in the oral-systemic health connection

Biomarkers of psychosocial stress were used in five studies (33%), with four studies (26%) finding a positive association between stress and oral and systemic diseases (72, 74, 79–81). Psychosocial stress was measured biologically using cortisol in salivary samples. Although other biomarkers of psychosocial stress were included in the search strategy, namely ACTH and brain-derived neurotrophic factor (BDNF), no studies assessing their association to oral-systemic health link were identified.

Salivary cortisol was correlated with greater clinical attachment loss, tooth loss, and periodontal disease in individuals with depression (74). Another study concluded that clinical attachment loss and mean cortisol levels were highest in individuals with both periodontitis and diabetes (72). A third article reported that salivary cortisol and DMFT (decayed, missing, and filled teeth) scores were higher in diabetic patients, and also found an association between salivary cortisol and the DMFT index (80).

The role of cortisol in periodontal disease severity among people with schizophrenia was not supported; although there were significantly higher values of periodontal disease parameters in people with schizophrenia, cortisol levels were much lower in cases compared to healthy controls (79). However, in individuals with Alzheimer's disease, higher cortisol levels and poorer oral health indicators were present compared to those with subjective cognitive decline or mild cognitive impairment (81).

## 4 Discussion

The aim of this scoping review was to map and identify gaps in the literature relating to the role of psychosocial factors in the co-occurrence of oral and systemic health conditions in the adult population. This review included a total of fifteen original research articles and focused on a range of both psychometric and biological measures of psychosocial stress. Among the psychometric indicators of stress were perceived stress, living conditions and lifestyle factors, and childhood adversity, in addition to biological measures of psychosocial stress using cortisol. The findings of the present review demonstrate an overall positive association between indicators of psychosocial stress with oral and systemic diseases, where higher levels of psychosocial stress were associated with a greater risk of both groups of conditions.

Previous research has identified stress as a premorbid factor associated with many risk factors for chronic disease (82, 83). Stress can stem from external events, such as major stressful life events or minor daily stressors, or from one's own perception of those experiences (84). Resilience and coping mechanisms aid in controlling emotions and changing the interaction between the person and the stressor, which reduces the stressor's physiologic effects (84–86). Adverse psychosocial factors in early childhood may also contribute to higher susceptibility to maladaptive stress responses that may manifest in adulthood since it can be imprinted on the developing brain (87, 88). For example, individuals who experience chronic stress as children often have a higher prevalence of systemic illnesses as adults, including heart disease (89) and autoimmune diseases (90). Despite the evidence on the crucial role of negative exposures in childhood on the risk of chronic disease later in life, we identified only one study that investigated the impact of psychosocial factors in childhood on oral health in relation to cardiovascular health and depression, thereby indicating a possible knowledge gap in this area.

We found the majority of studies included in this review assessed psychosocial stress in adults using psychometric measures. Exposure to psychosocial stress through various factors, including precarious social and living conditions in adulthood, takes a significant toll on biological mechanisms that are known to contribute to increased risk for oral and systemic health conditions. Our findings agree with other studies investigating the role of psychosocial stress in health outcomes. For example, low job control has been linked to an increased risk of all-cause and coronary heart disease mortality (91). Similarly, other factors, such as life satisfaction and social isolation, have been associated with a higher risk of stroke and transient ischemic attacks (92). On the contrary, social support has been found to be protective against dental pain (93), while individuals with greater perceived stress are more likely to report poor oral health (94).

In addition to psychometric measures of psychosocial stress, this review also investigated the association of the stress hormone cortisol as a biologic indicator of psychosocial stress with oral and systemic health. It is well established that higher levels of serum cortisol may be associated with psychosocial factors due to activation of the HPA axis (95–97). Cortisol is also engaged in additional long-term stress-related changes, such as regulating a number of physiological systems, including immunological responsiveness, in addition to short-term adaptive alterations. While it is normal for cortisol levels to fluctuate and remain elevated at specific times of the day (e.g., in the morning), when cortisol levels are persistently high throughout the day, this can be reflective of a the HPA axis hyperactivity (96, 97). In this review, we found the majority of the studies investigating the role of cortisol to support its association with both oral and systemic diseases. Examining other biomarkers that may be sensitive to the stress response can also provide new insights into the role of psychosocial stress in the development and progression of the oral-systemic disease link.

### 4.1 Strengths and limitations

The present study included fifteen observational studies from several countries reporting data of more than 50,000 participants with numerous psychometric and biological measures of psychosocial stress. Our review is the first to assess the role of psychosocial stress in oral and systemic disease conditions on a large scale while using both psychometric and biological measures of stress. This was done by following a systematic method for retrieving and analyzing the studies included in this scoping review. However, limitations of this study also exist. First, as our review included only studies published in English, we may have omitted relevant research written in other languages. Our results are also not reflective of the impact of psychosocial stress on oral and systemic health in children as this age group was excluded from our search strategy. However, with the exception of neurodevelopmental problems, systemic diseases are more common in adults and therefore including children may not have significantly altered our results. Future research and reviews should investigate the impact of psychosocial stress on oral and systemic health in the pediatric population.

## 5 Conclusion

Findings from the current scoping review suggest that biopsychosocial factors, as measured by psychometric and biological indicators, contribute to oral and systemic diseases. As an increasing proportion of the population grows older, it is integral to take a multidisciplinary approach to addressing social determinants of health, such as psychosocial stress, that contribute to disease. Our findings highlight the need to develop policies enhancing the common risk factors that lie within social and living conditions to mitigate the impact of oral-systemic disease in the population.

## Data Availability

The original contributions presented in the study are included in the article/**Supplementary Material**, further inquiries can be directed to the corresponding author.
