# Supplementary material for: Biopsychosocial factors in oral and systemic diseases: a scoping review
Source: Front Oral Health. 2024 May 30;5:1378467. doi: 10.3389/froh.2024.1378467 (PMC11169703; doi:10.3389/froh.2024.1378467)
Supplement: Supplementary file 1 [file Table1.docx]

**Supplemental File**

**Table S1.** Keyword search strategies for CINAHL, Embase, MEDLINE, and PsycINFO. For each database, keywords in each category were grouped together with “OR”, while the categories were grouped together with “AND”.

| **Database** | **Search strategy** |
| --- | --- |
| CINAHL | ((“oral health” OR “periodontal diseases” OR “periodontitis” OR “dental caries” OR “mouth diseases” OR “tooth loss” OR “tooth diseases”) AND (“chronic disease” OR “arthritis” OR “asthma” OR “cancer” OR “pulmonary disease, chronic obstructive” OR “dementia” OR “diabetes mellitus” OR “heart diseases” OR “mental disorders” OR “stroke”) AND (“stress, psychological” OR “psychological distress” OR “adverse childhood experiences” OR “biological markers” OR “hydrocortisone” OR “adrenocorticotropic hormone” OR “brain-derived neurotrophic factor”)) |
| Embase | ((“periodontal disease” OR “mouth disease” OR “periodontitis” OR “dental caries” OR “tooth disease”) AND (“chronic disease” OR “arthritis” OR “asthma” OR “chronic obstructive lung disease” OR “dementia” OR “diabetes mellitus” OR “heart disease” OR “mental disease” OR “cerebrovascular accident”) AND (“mental stress” OR “emotional stress” OR “childhood adversity” OR “childhood trauma” OR “early life stress” OR “life stress” OR “biological marker” OR “hydrocortisone” OR “corticotropin releasing factor” OR “brain-derived neurotrophic factor”)) |
| Medline | ((“oral health” OR “periodontal diseases” OR “periodontitis” OR “dental caries” OR “mouth diseases” OR “tooth loss” OR “tooth diseases”) AND (“arthritis” OR “asthma” OR “pulmonary disease, chronic obstructive” OR “dementia” OR “diabetes mellitus” OR “heart diseases” OR “mental disorders” OR “chronic disease” OR “stroke”) AND (“stress, psychological” OR “psychological distress” OR “adverse childhood experiences” OR “biomarkers” OR “hydrocortisone” OR “adrenocorticotropic hormone” OR “brain-derived neurotrophic factor”)) |
| PsycINFO | ((“oral health” OR “dental disorders” OR “dental health”) AND (“chronic illness” OR “arthritis” OR “asthma” OR “chronic obstructive pulmonary disease” OR “dementia” OR “diabetes mellitus” OR “heart disorders” OR “mental disorders” OR “cerebrovascular accidents”) AND (“psychological stress” OR “childhood adversity” OR “psychosocial factors” OR “biologic markers” OR “hydrocortisone” OR “corticotropin releasing factor” OR “brain-derived neurotrophic factor”)) |

**Table S2**. Reasons for full-text article exclusions.

| Ref # | Article | Reason for exclusion |
| --- | --- | --- |
| 1 | Ahmed et al. Stress and its predictors in pregnant women: A study in Saudi Arabia. Psychology Research and Behaviour Management. 2017;10:97-102. | No co-occurring oral-systemic disease assessment |
| 2 | Albandar JM. Global risk factors and risk indicators for periodontal diseases. Periodontol 2000. 2002;29:177-206. | Review |
| 3 | Arévalo-Flechas LC, Flores BP, Wang H, Liang H, Li Y, Gelfond J, Espinoza S, Lewis SL, Musi N, Yeh CK. Stress-Busting Program for Family Caregivers: Validation of the Spanish version using biomarkers and quality-of-life measures. Res Nurs Health. 2022;45(2):205-217. | Validation of psychological assessment |
| 4 | Axtelius BL, Söderfeldt BR. Oral disease and psychosocial risk determinants in relation to self-assessments of general health in persons with chronic whiplash-related disorders. Swed Dent J. 2003;27(4):185-95. | No systemic disease assessed |
| 5 | Bajaj DR, Khoso NA, Devrajani BR, Matlani BL, Lohana P. Oral lichen planus: a clinical study. J Coll Physicians Surg Pak. 2010;20(3):154-7. | No co-occurring oral-systemic disease assessment |
| 6 | Bensley L, VanEenwyk J, Ossiander EM. Associations of self-reported periodontal disease with metabolic syndrome and number of selfreported chronic conditions. Prev Chronic Dis 2011;8(3):A50. | No psychosocial stress exposure |
| 7 | Bissett J. How does diabetes affect oral health? Dental Nursing. 2017;13(1):32-3. | Editorial/Opinion |
| 8 | Cakmak O, Alkan BA, Ozsoy S, Sen A, Abdulrezzak U. Association of gingival crevicular fluid cortisol/dehydroepiandrosterone levels with periodontal status. J Periodontol. 2014;85(8):e287-94. | No co-occurring oral-systemic disease assessment |
| 9 | Candelaria LM, Huttula CS. Angioedema associated with angiotensin-converting enzyme inhibitors. J Oral Maxillofac Surg. 1991;49(11):1237-9. | Review |
| 10 | Carreira-Míguez M, Navarro-Jiménez E, Clemente-Suárez VJ. Behavioral Patterns of Depression Patients and Control Population. Int J Environ Res Public Health. 2022;19(15):9506. | No co-occurring oral-systemic disease assessment |
| 11 | Chatzopoulou E, Rangé H, Deraz O, Boutouyrie P, Perier MC, Guibout C, Thomas F, Andrieu M, Bailly K, Vedie B, Danchin N, Jouven X, Bouchard P, Empana JP. Poor Masticatory Capacity and Blood Biomarkers of Elevated Cardiovascular Disease Risk in the Community: The Paris Prospective Study III. Arterioscler Thromb Vasc Biol. 2021;41(7):2225-2232. | No co-occurring oral-systemic disease assessment |
| 12 | Chen JJ, Liu LF, Lin CI, Lin HC. Multidimensional Determinants of Well-Being Among Community-Dwelling Older Adults During the Early Stage of the COVID-19 Pandemic in Taiwan. Gerontol Geriatr Med. 2022;8:23337214221111227. | COVID-19 |
| 13 | Cheshmedzhieva AY, Totomirova TT, Arnaudova MV, Grigorov NB, Naseva EK. Specific dental treatment algorithm in patients with diabetes mellitus. Endocrinologia Jrnl. 2020 | Not in English |
| 14 | DeBate R, Daley EM, Vamos CA, Kline N, Marsh L, Smith S. Transdisciplinary women's health: a call to action. Health Care Women Int. 2014;35(10):1113-32. | Editorial/Opinion |
| 15 | Decker A, Askar H, Tattan M, Taichman R, Wang HL. The assessment of stress, depression, and inflammation as a collective risk factor for periodontal diseases: a systematic review. Clin Oral Investig. 2020;24(1):1-12. | Review |
| 16 | Degerlund Maldi K, San Sebastian M, Gustafsson PE, Jonsson F. Widespread and widely widening? Examining absolute socioeconomic health inequalities in northern Sweden across twelve health indicators. Int J Equity Health. 2019;18(1):197. | No co-occurring oral-systemic disease assessment |
| 17 | Deng Y, He S, Wang J. Validation of the Hospital Anxiety and Depression Scale and the Perceived Stress Scale and psychological features in patients with periodontitis. J Periodontol. 2021;92(11):1601-1612. | Validation of psychological assessment No co-occurring oral-systemic disease assessment |
| 18 | Di Stasio D, Candotto V, Serpico R, Migliozzi R, Petruzzi M, Tammaro M, Maio C, Gritti P, Lauritano D, Lucchese A. Depression and distress in burning mouth syndrome: A case control study. J Biol Regul Homeost Agents. 2018;32(2 Suppl. 1):91-95. | Systemic disease assessed as psychological stress No co-occurring oral-systemic disease assessment |
| 19 | Dumitrescu AL, Gârneaţă L, Guzun O. Anxiety, stress, depression, oral health status and behaviours in Romanian hemodialysis patients. Rom J Intern Med. 2009;47(2):161-8. | No psychological stress exposure to co-occurring oral-systemic disease assessment |
| 20 | Eadie MJ. The management of facial pain. Med J Aust. 1975 Aug 9;2(6):224-5. | Review |
| 21 | Eishi A. Frequency of Cancers and Their Demographic Characteristics in Patients Referred to Omid Hospital of Urmia. International Journal of Tropical Medicine, 2016;11:13-20. | No psychological stress exposure or co-occurring oral-systemic disease assessment |
| 22 | Eli I, Baht R, Littner MM, Kleinhauz M. Detection of psychopathologic trends in glossodynia patients. Psychosom Med. 1994;56(5):389-94. | No co-occurring oral-systemic disease assessment |
| 23 | Fehlberg BK, Barros MBA, Lima MG. Health behaviors and multimorbidity associated with bruxism: Population-based study. Oral Dis. 2023;29(1):245-253. | No psychological stress exposure to co-occurring oral-systemic disease assessment |
| 24 | Friedlander AH, Norman DC. Late-life depression: psychopathology, medical interventions, and dental implications. Oral Surg Oral Med Oral Pathol Oral Radiol Endod. 2002;94(4):404-12 | Review |
| 25 | Giuca MR, Miceli M, Carli E, Lardani L, Marchio V, Baldini C. Impact of Sjögren's syndrome on oral health and quality of life: an observational cross-sectional study. J Biol Regul Homeost Agents. 2020;34(3 Suppl. 1):129-137. | No psychosocial stress exposure |
| 26 | Goud S, Chandra RV, Reddy AA, Reddy BH, Nagarajan S, Naveen A. Possible Association Between the Mallampati Score of the Oropharynx and Measures of Tongue Coating, Oral Hygiene and Periodontal Status. Oral Health Prev Dent. 2016;14(5):423-432. | No psychosocial stress exposure |
| 27 | Hugo FN, Hilgert JB, Bozzetti MC, Bandeira DR, Gonçalves TR, Pawlowski J, de Sousa Mda L. Chronic stress, depression, and cortisol levels as risk indicators of elevated plaque and gingivitis levels in individuals aged 50 years and older. J Periodontol. 2006;77(6):1008-14. | No psychological stress exposure to co-occurring oral-systemic disease assessment |
| 28 | Iskander S, Samim F. Patient Characteristics Associated with the Development of Oral Lichen Planus in Two Provinces in Canada. J Can Dent Assoc. 2022;88:m5. | No psychological stress exposure or co-occurring oral-systemic disease assessment |
| 29 | Johannsen A, Rydmark I, Söder B, Asberg M. Gingival inflammation, increased periodontal pocket depth and elevated interleukin-6 in gingival crevicular fluid of depressed women on long-term sick leave. J Periodontal Res. 2007;42(6):546-52. | No psychological stress exposure to co-occurring oral-systemic disease assessment |
| 30 | Kabani F, Lykens K, Tak HJ. Exploring the relationship between adverse childhood experiences and oral health-related quality of life. J Public Health Dent. 2018;78(4):313-320. | No co-occurring oral-systemic disease assessment |
| 31 | Kirkengen AL, Lygre H. Exploring the relationship between childhood adversity and oral health: An anecdotal approach and integrative view. Med Hypotheses. 2015;85(2):134-40. | Review |
| 32 | Klages U, Weber AG, Wehrbein H. Approximal plaque and gingival sulcus bleeding in routine dental care patients: relations to life stress, somatization and depression. J Clin Periodontol. 2005;32(6):575-82. | No co-occurring oral-systemic disease assessment |
| 33 | Kurushima Y, Bowyer R, Ide M, Hughes FJ, Steves CJ. Genetic and environmental contributions to the association between mood disorder and periodontal disease: A cross-sectional study among female twins in the UK. J Clin Periodontol. 2019;46(1):40-50. | No psychological stress exposure to co-occurring oral-systemic disease assessment |
| 34 | Laforgia A, Corsalini M, Stefanachi G, Pettini F, Di Venere D. Assessment of Psychopatologic Traits in a Group of Patients with Adult Chronic Periodontitis: Study on 108 Cases and Analysis of Compliance during and after Periodontal Treatment. Int J Med Sci. 2015;12(10):832-9. | No co-occurring oral-systemic disease assessment |
| 35 | Matear DW, Locker D, Stephens M, Lawrence HP. Associations between xerostomia and health status indicators in the elderly. J R Soc Promot Health. 2006;126(2):79-85. | No psychosocial stress exposure |
| 36 | Mohamadi Hasel K, Besharat MA, Abdolhoseini A, Alaei Nasab S, Niknam S. Relationships of personality factors to perceived stress, depression, and oral lichen planus severity. Int J Behav Med. 2013;20(2):286-92. | No co-occurring oral-systemic disease assessment |
| 37 | Moss ME, Beck JD, Kaplan BH, Offenbacher S, Weintraub JA, Koch GG, Genco RJ, Machtei EE, Tedesco LA. Exploratory case-control analysis of psychosocial factors and adult periodontitis. J Periodontol. 1996;67(10 Suppl):1060-9. | No co-occurring oral-systemic disease assessment |
| 38 | Ng SK, Keung Leung W. A community study on the relationship between stress, coping, affective dispositions and periodontal attachment loss. Community Dent Oral Epidemiol. 2006;34(4):252-66. | No co-occurring oral-systemic disease assessment |
| 39 | Noguchi S, Makino M, Haresaku S, Shimada K, Naito T. Insomnia and depression impair oral health-related quality of life in the old-old. Geriatr Gerontol Int. 2017;17(6):893-897. | No psychosocial stress exposure |
| 40 | Peltzer K, Pengpid S. Angina pectoris among middle-aged and older adults in India: Prevalence and correlates from a national cross-sectional survey in 2017-2018. International Journal on Disability and Human Development. 2022;21(1):65-72. | No oral health outcome |
| 41 | Pengpid S, Peltier K. Prevalence and correlates of angina pectoris among a nationally representative population-based sample of older adults in Mexico. In Mexico: Health and Aging. Nova Science Publishers, Inc. 2020. p. 107-118 | No oral health outcome |
| 42 | Persson GR. What has ageing to do with periodontal health and disease? Int Dent J. 2006;56(4 Suppl 1):240-9. | Review |
| 43 | Pires ALPV, Alves LDB, da Silva AM, Arsati F, Lima-Arsati YBO, Dos Santos JN, Calumby RT, Freitas VS. Salivary biomarkers to evaluate psychological disorders in oral lichen planus: A systematic review with meta-analysis. Oral Dis. 2023;29(7):2734-2746. | Review |
| 44 | Reynolds MA. Modifiable risk factors in periodontitis: at the intersection of aging and disease. Periodontology 2000. 2014;64(1):7-19. | Review |
| 45 | Ritter AV. High blood pressure and oral health. J Esthet Restor Dent. 2007;19(2):125-6. | Editorial/Opinion |
| 46 | Saalim M, Sansare K, Karjodkar FR, Ali IK, Sharma SR, Kapoor R, Mehra A, Rahman B. Oral submucous fibrosis and its impact on psychological stress: a case-control study. Psychol Health Med. 2022;27(4):735-745. | No psychosocial stress exposure |
| 47 | Sabbah W, Watt RG, Sheiham A, Tsakos G. Effects of allostatic load on the social gradient in ischaemic heart disease and periodontal disease: evidence from the Third National Health and Nutrition Examination Survey. J Epidemiol Community Health. 2008;62(5):415-20. | No co-occurring oral-systemic disease assessment |
| 48 | Senba T, Kobayashi Y, Inoue K, Kaneto C, Inoue M, Toyokawa S, Suyama Y, Suzuki T, Miyano Y, Miyoshi Y. The association between self-reported periodontitis and coronary heart disease--from MY Health Up Study--. J Occup Health. 2008;50(3):283-7. | No psychological stress exposure to co-occurring oral-systemic disease assessment |
| 49 | Shah B, Ashok L, Sujatha GP. Evaluation of salivary cortisol and psychological factors in patients with oral lichen planus. Indian J Dent Res. 2009;20(3):288-92. | No co-occurring oral-systemic disease assessment |
| 50 | Sokolovic S, Dagher S, Dautbegovic A, Jamal E. Major stress linked to pathogenesis of rheumatoid arthritis - A case report. Bone Reports. 2020;13:100512, | Case-report |
| 51 | Solis AC, Lotufo RF, Pannuti CM, Brunheiro EC, Marques AH, Lotufo-Neto F. Association of periodontal disease to anxiety and depression symptoms, and psychosocial stress factors. J Clin Periodontol. 2004;31(8):633-8. | No psychological stress exposure to co-occurring oral-systemic disease assessment |
| 52 | Spangler JG, Konen JC. Oral health behaviors in medical patients with diabetes mellitus. J Dent Hyg. 1994;68(6):287-93. | No psychological stress exposure to co-occurring oral-systemic disease assessment |
| 53 | Stabholz A, Soskolne WA, Shapira L. Genetic and environmental risk factors for chronic periodontitis and aggressive periodontitis. Periodontol 2000. 2010;53:138-53. | Review |
| 54 | Svensson P, Kaaber S. General health factors and denture function in patients with burning mouth syndrome and matched control subjects. J Oral Rehabil. 1995;22(12):887-95. | No psychological stress exposure to co-occurring oral-systemic disease assessment |
| 55 | Tabolli S, Bergamo F, Alessandroni L, Di Pietro C, Sampogna F, Abeni D. Quality of life and psychological problems of patients with oral mucosal disease in dermatological practice. Dermatology. 2009;218(4):314-20. | No psychological stress exposure to co-occurring oral-systemic disease assessment |
| 56 | Ueno M, Izumi Y, Kawaguchi Y, Ikeda A, Iso H, Inoue M, Tsugane S; JPHC Study Group. Prediagnostic plasma antibody levels to periodontopathic bacteria and risk of coronary heart disease. Int Heart J. 2012;53(4):209-14. | No oral health outcome |
| 57 | Vasiliou A, Shankardass K, Nisenbaum R, Quiñonez C. Current stress and poor oral health. BMC Oral Health. 2016;16(1):88. | No co-occurring oral-systemic disease assessment |
| 58 | Vettore MV, Leão AT, Monteiro Da Silva AM, Quintanilha RS, Lamarca GA. The relationship of stress and anxiety with chronic periodontitis. J Clin Periodontol. 2003;30(5):394-402. | No co-occurring oral-systemic disease assessment |
| 59 | Vettore M, Quintanilha RS, Monteiro da Silva AM, Lamarca GA, Leão AT. The influence of stress and anxiety on the response of non-surgical periodontal treatment. J Clin Periodontol. 2005;32(12):1226-35. | No co-occurring oral-systemic disease assessment |
| 60 | Vitaliano PP, Persson R, Kiyak A, Saini H, Echeverria D. Caregiving and gingival symptom reports: psychophysiologic mediators. Psychosom Med. 2005;67(6):930-8. | No psychological stress exposure or co-occurring oral-systemic disease assessment |
| 61 | Warren KR, Postolache TT, Groer ME, Pinjari O, Kelly DL, Reynolds MA. Role of chronic stress and depression in periodontal diseases. Periodontol 2000. 2014;64(1):127-38. | Review |
| 62 | Weatherspoon DJ, Borrell LN, Johnson CW, Mujahid MS, Neighbors HW, Adar SD. Racial and Ethnic Differences in Self-Reported Periodontal Disease in the Multi-Ethnic Study of Atherosclerosis (MESA). Oral Health Prev Dent. 2016;14(3):249-57. | No co-occurring oral-systemic disease assessment |
| 63 | Wellappulli N, Ekanayake L. Association between psychological distress and chronic periodontitis in Sri Lankan adults. Community Dent Health. 2019;36(4):293-297. | No co-occurring oral-systemic disease assessment |
| 64 | Zhang H, Chen B, Pan C, Zhang A. To evaluate the serum cortisol, salivary cortisol, and serum interleukin-1 B level in patients of chronic periodontitis with smoking and stress and without smoking and stress. Medicine (Baltimore). 2021;100(31):e26757. | No co-occurring oral-systemic disease assessment |
| 65 | Zhu L, Zhou C, Chen S, Huang D, Jiang Y, Lan Y, Zou S, Li Y. Osteoporosis and Alveolar Bone Health in Periodontitis Niche: A Predisposing Factors-Centered Review. Cells. 2022;11(21):3380. | Review |
